# Impact of Breed and Slaughter Hygiene on Beef Carcass Quality Traits in Northern Greece

**DOI:** 10.3390/foods14101776

**Published:** 2025-05-16

**Authors:** Vasiliki Papanikolopoulou, Anestis Tsitsos, Stella Dokou, Stergios Priskas, Sotiria Vouraki, Vangelis Economou, Ioanna Stylianaki, Angeliki Argyriadou, Georgios Arsenos

**Affiliations:** 1 Laboratory of Animal Production and Environmental Protection, School of Veterinary Medicine, Faculty of Health Sciences, Aristotle University of Thessaloniki, 54124 Thessaloniki, Greece; vipapani@vet.auth.gr (V.P.); stpriskas@vet.auth.gr (S.P.); svouraki@uoi.gr (S.V.); argyrian@vet.auth.gr (A.A.); 2 Laboratory of Animal Food Products Hygiene—Veterinary Public Health, School of Veterinary Medicine, Faculty of Health Sciences, Aristotle University of Thessaloniki, 54124 Thessaloniki, Greece; tsitanes@vet.auth.gr (A.T.); boikonom@vet.auth.gr (V.E.); 3 Laboratory of Animal Nutrition, School of Veterinary Medicine, Faculty of Health Sciences, Aristotle University of Thessaloniki, 54124 Thessaloniki, Greece; dokoustella@vet.auth.gr; 4 Laboratory of Animal Production, Nutrition and Biotechnology, Department of Agriculture, School of Agriculture, University of Ioannina, 47100 Arta, Greece; 5 Laboratory of Pathology, School of Veterinary Medicine, Faculty of Health Sciences, Aristotle University of Thessaloniki, 54124 Thessaloniki, Greece; stylioan@vet.auth.gr

**Keywords:** cattle, meat, physicochemical characteristics, microbiological analyses, muscle fiber

## Abstract

The objective of this study was to assess the impact of breed and slaughter hygiene practices on beef quality traits in Northern Greece. A random sample of 159 beef carcasses from three breeds, Aberdeen Angus (AA, n = 38), Holstein (HO, n = 42), and Limousin (LI, n = 40), and crossbred (CR, n = 39) males were used. The chroma, pH, texture, chemical composition, and fatty acid profile were assessed using the *Longissimus dorsi* muscle. The muscle histomorphometry was assessed using the psoas major samples. Microbiological analyses were conducted on the beef carcasses to evaluate slaughter hygiene. A comparative analysis using ANOVA, Mann–Whitney, and Kruskal–Wallis tests was performed to assess the effects of breed and slaughter hygiene on the meat quality traits. The meat quality differed significantly (*p* < 0.05) between the breeds. Specifically, the LI beef exhibited higher lightness (L*) values compared to those of the AA and HO beef. The CR breed produced the reddest beef, which differed significantly from the AA and HO beef. The beef yellowness (b*) was higher in the AA breed compared to the other breeds. The AA and CR beef was more tender than the LI beef. The AA beef exhibited the lowest protein and highest fat content, while the LI beef was the leanest. The monounsaturated fatty acid (MUFA) concentrations in the AA beef were 22% higher than those in the HO beef, whereas the HO beef had 23% higher levels of saturated fatty acids (SFAs). The total mesophilic viable counts among the slaughterhouses exceeded the lower acceptable threshold (3.5 log CFU/cm^2^), indicating inadequate slaughter hygiene practices that could impact beef quality and safety. Globally, this is the first comprehensive study that uniquely combines techniques for assessing beef quality from whole carcasses to individual muscle fibers.

## 1. Introduction

Globally, beef is the third most consumed meat, accounting for 21% of the total meat consumption, following poultry (38%) and pork (34%) [1]. Projections indicate that by 2033, poultry and pork consumption will grow by 16% and 8%, respectively, while beef consumption is expected to increase by 11%, underscoring its continued importance in global diets [2]. The demand for beef is driven by its high nutritional value, offering high-quality protein and essential vitamins and minerals like heme iron, zinc, B vitamins, retinol, and selenium. Red meat also contains beneficial fatty acids, including monounsaturated and polyunsaturated fats, contributing to overall human health [3,4].

Global beef and veal production has remained relatively stable, fluctuating between 57 and 60 million metric tons annually from 2012 to 2024. The United States is the leading producer, accounting for 20% of global production, followed by Brazil (18%), China (13%), and the European Union (EU) (11%). Within the EU, the total beef production reached 6.4 million tonnes in 2023, with France (20.7%), Germany (17.3%), Ireland (10.9%), Italy (9.7%), Poland (9.3%), and Spain (9.1%) being the primary contributors. Major veal producers within the EU are the Netherlands (27.5%), Spain (21.9%), France (18.3%), and Italy (9.6%) [5].

The bovine population in Greece totals 622,400 heads, encompassing dairy cattle, beef cattle, and buffaloes, reared across extensive, semi-intensive, and intensive farming systems [6]. In northern Greece, Central Macedonia is the country’s leading cattle farming region (24%), comprising 1605 farms, 55% of which are classified as commercial operations with over 50 animals [7]. Cattle raised for fattening exhibit significant genetic diversity, including local breeds (Brachyceros, Katerini, and Sykia), foreign breeds (Limousin, Black Angus, Holstein, Belgian Blue, Charolais, Simmental, Baltata Romaneasca, Aubrac, and Blonde d’ Aquitaine), and their crosses [8].

Beef consumption in Greece reaches 14.8 kg of meat per capita annually, surpassing the EU-27 average (14.0 kg annually) [1]. Consumption is shaped by safety and quality, with safety being a prerequisite and quality reflecting consumer preferences [9]. Beef quality is a key driver in consumer purchasing preferences, with quality traits like colour, fat content, tenderness, and juiciness playing a significant role [10]. These quality traits can be objectively evaluated in laboratory settings, providing a more precise and standardized assessment of beef quality [11]. Meat quality, however, is influenced by numerous intrinsic (animal-related, such as breed, age, and sex) and extrinsic factors (environmental-related, including feeding, farming system, and slaughter procedures) [12,13]. Among these, breed is a key factor in assessing meat quality, with notable differences reported in tenderness, juiciness, and flavour intensity among various breeds. These variations in meat quality traits between breeds are likely attributed to different management practices across countries [14]. Generally, the early-maturing AA breed has been associated with higher intramuscular fat (IMF) and received better sensory ratings than late-maturing HO, LI, and Charolais breeds [15]. Furthermore, slaughter procedures can significantly affect the microbiological quality of carcasses. If good hygiene practices are not met, this may lead to a reduced shelf life and compromised meat quality. Therefore, ensuring the high microbiological quality of beef is important for consumer satisfaction and food safety [11].

Numerous studies have compared meat quality from various cattle breeds and assessed slaughter hygiene across different slaughterhouses [15,16,17,18]. Similarly, these factors are also likely to influence the quality of meat produced in Greece. However, relevant studies within the country remain scarce. Furthermore, a comprehensive analysis of beef quality traits across multiple stages of the production process, from whole carcasses to individual muscle fibers, is notably absent from the global literature. Conducting such research is crucial for collecting valuable data that could benefit breeders, the meat industry, and slaughterhouses to adopt effective strategies to meet the growing consumer demand for high-quality and safe products. Hence, this study aims to address this gap by assessing the impact of breed and slaughter hygiene practices on meat quality traits in Northern Greece.

## 2. Materials and Methods

### 2.1. Study Area, Animals and Abattoirs

A random sample of 120 purebred males of three breeds was included in this study, namely, Aberdeen Angus (AA, n = 38), Holstein (HO, n = 42), and Limousin (LI, n = 40), along with 39 crossbred males (CR), most of which were Charolais x Limousin crosses. All animals were reared under intensive systems, housed in feedlots, pens (6–9 animals per pen) with concrete flooring. They were all provided with a high-concentrate, grain-based diet formulated to meet their nutritional requirements. The diet primarily consisted of cereal grains (maize and barley), soybean meal, wheat bran, a vitamin–mineral supplement, with maize silage and straw served as the roughage source. No additional feed additives were used on any of the farms. Feed was administered twice daily. Cattle were fed ad libitum. Fresh water was also available ad libitum through automatic drinkers in each pen. Animals were slaughtered at three different commercial abattoirs in Northern Greece between the summer of 2019 and the summer of 2024. Each facility is licensed to slaughter ruminants and pigs and operates two distinct slaughter lines. The cattle slaughter line is entirely separate, while the slaughter of small ruminants and pigs is carried out in parallel. All abattoirs were located in lowland areas and operated in accordance with Regulation (EC) No 853/2004 [19]. Average age at slaughter was 24.3 ± 3.44, 17.0 ± 1.43, 20.3 ± 2.42, and 18.4 ± 3.50 months for AA, HO, LI, and CR males, respectively. Data used in this study were collected within four different projects: Marbling Meat—Development of innovative breeding methods to control the quantity, quality and distribution of intramuscular fat in intensively fed cattle”; BlackWhite—“Production and fattening of black and white calves in dairy cows”; BQM—“Development of a special feeding method of beef cattle and implementation of a total quality approach from stable to table”; and GreQuM—“Innovative technologies to increase the competitiveness of Greek meat: Greek Quality Meat”.

### 2.2. Physicochemical Analyses

Meat samples were obtained from beef carcasses following the methodology outlined by Tsitsos et al. [11]. Approximately 200 g of meat was excised from the 13th rib of each carcass. The samples were transported to the laboratory in insulated polystyrene boxes maintained at ≤4 °C and stored in vacuum packaging under refrigeration (≤4 °C) for further analysis. On the first day after slaughter, samples were subjected to physicochemical analyses, including pH measurements, colorimetry, texture profile analysis (TPA), chemical composition (moisture, total fat and protein, collagen), and fatty acid profile.

#### 2.2.1. Meat pH

Meat pH measurement was performed using a portable pH meter (FiveGo pH meter F2, Mettler Toledo, Zaventem, Belgium). The measurement was performed non-destructively by inserting the electrode into a small incision in each sample. To ensure reliability, three consecutive records were taken at the same site, and the average value was calculated. The pH meter was calibrated prior to use with two standard buffer solutions at pH 4.00 and pH 7.00 to ensure measurement accuracy.

#### 2.2.2. Meat Colour

Meat colour assessment was conducted following the methodology outlined by Tsitsos et al. [20]. Colour measurements were taken of freshly exposed meat surfaces immediately after unpacking. A Konica Minolta CR-410 Chroma Meter (Tokyo, Japan) was used for analysis, configured with a 50 mm aperture, illuminant C, and a 2° observer angle. The instrument was calibrated using a white reference tile with values of Y: 94.8, X: 0.3130, and y: 0.3190. Each sample was scanned three times at different positions, aligned perpendicular to the muscle fibers, while avoiding areas with visible fat or connective tissue. The recorded values for lightness (L*), redness (a*), and yellowness (b*) were averaged for each sample. In addition, chroma and hue angle were calculated using the formulas recommended by the American Meat Science Association (AMSA) [21]:

Chroma = (a*^2^ + b*^2^)^1/2^

Hue angle = arctangent (b*/a*)

#### 2.2.3. Meat Texture

Texture profile analysis (TPA) was conducted using a Stable Micro Systems TA.HD plus Texture Analyser (Godalming, UK) equipped with a flat-faced cylindrical probe (1.27 cm diameter). The analyzer was operated via Exponent software (version 6.1.16.0). Uniformly sized, oval-shaped samples (2–3 cm in width and thickness) were excised from the center of each meat piece for analysis. A double-compression cycle test was applied, with the probe compressing the sample perpendicularly to the muscle fibers. Test parameters included a pre-test speed of 1.00 mm/s, a test speed of 5.00 mm/s, and a post-test speed of 5.00 mm/s, achieving 40% deformation of the sample height in each cycle. The time interval between two compressions was set at 2.02 s. The force–time plots generated during the analysis represented the sample’s resistance to compression over time, enabling the calculation of key texture parameters such as Hardness 1, Hardness 2, Cohesiveness, Springiness, and Chewiness, as described by Skaperda et al. [22].

#### 2.2.4. Meat Chemical Composition and Fatty Acid Profile

Meat chemical composition was analyzed according to the methodology described by Tsitsos et al. [23]. For the analysis, 100 g of meat was weighed and placed in a plastic sample pan for evaluation using a near-infrared spectrometer (NIR, Perten DA7250, Perkin Elmer Ltd., Waltham, MA, USA) calibrated for meat and meat products. The calibration process followed ISO- and AOAC-approved protocols, including Soxhlet extraction for fat, the Kjeldahl method for protein, and hydroxyproline analysis for collagen. Calibration models were developed using Artificial Neural Networks (ANNs) and Honigs Regression™ to ensure the precision and reliability of the measurements.

The fatty acid profile of beef samples was analyzed using the Soxtherm Soxhlet Extraction System (C. Gerhardt GmbH & Co. KG, Königswinter, Germany), in compliance with AOAC Method 991.36, as described by Tsitsos et al. [23]. The extracted fatty acids were transesterified in a methanolic potassium hydroxide solution to produce fatty acid methyl esters (FAMEs, Supelco 37 Component FAME Mix CRM47885, Merck KGaA, Darmstadt, Germany), which were then analyzed by gas chromatography with flame ionization detection (GC-FID). The chromatographic analyses were performed using a Shimadzu GC-2010 Plus gas chromatography system, equipped with an FID detector and a Supelco SP2560 column (100 m × 0.25 mm × 0.20 μm). Helium (purity 99.999%) was used as the carrier gas at a flow rate of 2 mL/min. The injection volume was 1 μL with a split ratio of 1:50, and the injector and detector temperatures were maintained at 250 °C. The oven temperature program started at 110 °C (held for 7 min) and increased at 3 °C/min to 190 °C (held for 2 min). It then rose at 0.5 °C/min to 205 °C, followed by a 5 °C/min rise to 230 °C (maintained for 5 min), and a final step to 240 °C at 5 °C/min, held for another 5 min. The total runtime for the analysis was 82.67 min.

### 2.3. Muscle Histomorphometry

Muscle histomorphometry was conducted following the protocol outlined by Argyriadou et al. [24] in LI, CR, and HO breeds. Briefly, samples of the psoas major muscle were collected from the carcasses, fixed in formalin, embedded in paraffin, sectioned at 4 μm, and stained with hematoxylin and eosin. In order to perform morphometric analysis, images of muscle cross-sections were captured at 10× magnification, with 15 images taken per sample. From these, two to eight images were randomly selected for analysis, yielding a total of 1008 to 1692 muscle fibers per animal. The minimum Feret’s diameter of each muscle fiber was automatically calculated for each image. The morphometric analysis was carried out using the “analyze particles” function in ImageJ software (J-4 Version 1.54g, NIH, Bethesda, MD), following the methodology described by Varian et al. [25].

### 2.4. Microbiological Analysis

Microbiological analysis was performed on samples collected from two of the three slaughterhouses included in the study to assess the hygienic status of produced carcasses and to compare the slaughter hygiene between two abattoirs operating under different slaughtering conditions. Specifically, at the time of the study, abattoir A slaughtered only bovine animals at a relatively low throughput (2000 animals annually), whereas abattoir B operated at a higher capacity (17,000 bovine per year) and slaughtered pigs and small ruminants in addition to bovine animals. Surface sampling of each carcass was performed approximately one hour after slaughter using the swab method. A sterile sponge soaked in 10 mL of Maximum Recovery Diluent (MRD, Oxoid Ltd., Basingstoke, UK) and a dry sterile sponge were used to swab the forequarter, hindquarter, and abdominal areas of each carcass. The sponges were then aseptically placed in a stomacher bag (Interscience, Saint-Nom-la-Bretêche, France) and transported to the laboratory in a portable refrigerator (≤4 °C) within two hours of collection. Within 24 h of sampling, the stomacher bags with the sponges were brought to room temperature (20 °C), filled with 100 mL of MRD, and homogenized in a stomacher (Lab Blender, Interscience) for two minutes. Decimal dilutions were subsequently prepared in MRD-containing tubes. The analyses included total mesophilic viable counts (TMVCs) and total psychrophilic viable counts (TPVCs) according to ISO 4833-1:2013 [26], as well as coliform counts in accordance with ISO 21528-2:2017 [27], as outlined in Commission Regulation (EC) No. 2073/2005 on microbiological criteria for foodstuffs [28]. From each dilution, 1 mL was surface-inoculated onto the appropriate media. Enumeration of TMVCs and TPVCs was conducted on Plate Count Agar (Biolab Diagnostics, Budapest, Hungary), while Violet Red Bile Agar (Biolab Diagnostics) was used for the coliform counts. The incubation conditions were 30 °C for 72 h 10 °C for 7 days, and 37 °C for 24 h for TMVCs, TPVCs, and coliform counts, respectively. After incubation, the characteristic colonies were counted, and the results were recorded.

### 2.5. Statistical Analysis

All analyses were performed using R Statistical Software (v4.1.2; R Core Team 2021) [29]. Descriptive statistics were calculated using “psych” and “dplyr” statistical packages [30,31]. A one-way analysis of variance (ANOVA) was performed to examine the relationship between age and breed. A significant association (*p* < 0.05) between these factors was observed. Given this strong collinearity, age was not included in our final model as it was considered a confounding factor. The Shapiro–Wilk test was used to assess whether the data followed a normal distribution. The effect of breed on normally distributed data (lightness—L*, redness—a*, yellowness—b*, and chroma) was assessed using ANOVA. Post hoc comparisons between breeds were conducted using Tukey’s and Duncan’s test. Levene’s test was applied to assess the homogeneity of variances. Data that did not follow a normal distribution were assessed using either the Mann–Whitney test (for comparisons involving two groups, such as the microbiological results between the two slaughterhouses) or the Kruskal–Wallis test (for comparisons involving three or more groups, such as the BA, LI, HO, and CR groups). Post hoc comparisons for non-normally distributed data were carried out using Dunn’s test with the Bonferroni correction to detect statistically significant differences between groups. In all cases, the level of statistical significance was set at 0.05.

## 3. Results

### 3.1. Descriptive Statistics and Effects of Breed (Aberdeen Angus (AA), Holstein (HO), Limousin (LI) Breed, Crossbred (CR) Males) on Carcass Quality Traits

#### 3.1.1. Descriptive Statistics

Descriptive statistics for pH, colour and texture parameters, chemical composition, fatty acid profile, and Feret’s diameter per breed carcasses are presented in Table 1. Additionally, all raw data are provided in Supplementary Table S1. Overall, the average values of the studied traits showed slight variation within each breed.

#### 3.1.2. Effects of Breed on Meat pH, Colour, and Texture Parameters

The effects of breed on the meat pH, colour, and texture parameters are presented in Table 2 and Table 3. The beef produced by the LI breed was significantly (*p* < 0.001) paler (higher L*) compared to the AA and HO beef. The CR males produced the reddest beef (higher a*), which differed significantly (*p* < 0.001) from the AA and HO beef. The AA and CR beef exhibited higher and lower b* values, respectively, which differed significantly (*p* < 0.001) from all the other groups included in the study. Furthermore, the HO beef exhibited intermediate b* values that also differed significantly (*p* < 0.001) from those of the AA and CR beef. Regarding chroma, the HO beef exhibited the lowest values (*p* < 0.001) compared to the other breeds. Additionally, the hue angle differed significantly (*p* < 0.001) between the breeds.

The tenderness was also significantly affected by the breed. Specifically, the LI beef was less (*p* < 0.001) tender compared to the AA, CR, and HO beef. Regarding Springiness, the LI beef had higher (*p* < 0.001) values compared to those of the HO and CR beef, while significant differences were also found between the AA and CR beef. The meat cohesiveness differed significantly (*p* < 0.05) between the AA and LI breeds. Higher (*p* < 0.001) values of Chewiness were observed for the LI beef compared to those for the AA, CR, and HO beef. The effect of breed on the meat pH_24_ was not statistically significant (*p* > 0.05)_._

#### 3.1.3. Effects of Breed on Meat Chemical Composition and Fatty Acid Profile

The effects of the breed on the meat chemical composition and fatty acid profile are presented in Table 4 and Table 5, respectively. The meat protein content was significantly (*p* < 0.001) lower for the AA breed, compared to the LI and HO breeds, which had similar values (*p* > 0.05). In addition, the AA beef had the highest fat levels (*p* < 0.001), while the LI breed produced the leanest meat (*p* < 0.001). The collagen concentration was lower (*p* < 0.001) in the LI and HO beef compared to the AA beef. Regarding the fatty acid profile, the AA beef exhibited higher (*p* < 0.001) MUFA concentrations, including myristoleic, palmitoleic, and oleic acid, compared to those of the HO beef. Furthermore, the HO beef had higher (*p* < 0.001) levels of total saturated fatty acids (SFAs), particularly palmitic and stearic acid, compared to the AA beef. Although the PUFA concentrations did not differ significantly among the breeds (*p* > 0.05), the linoleic acid levels were notably higher (*p* < 0.001) in the HO beef.

#### 3.1.4. Effect of Breed on Muscle Histomorphometry

**Figure 1:**
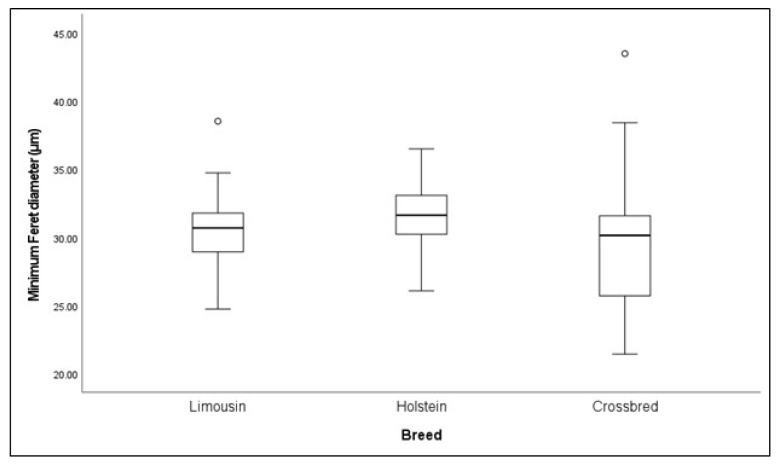
Box plots of minimum Feret diameter among Limousin (LI), Holstein (HO), and crossbred (CR) males.

The effect of breed on the muscle fiber minimum Feret diameter is presented in Figure 1. The minimum Feret diameter did not differ significantly among the examined cattle breeds (*p* > 0.05).

### 3.2. Slaughter Hygiene Assessment

The microbiological analyses results are presented in Table 6. Significant differences in the microbial counts for the beef carcasses were observed between the two examined slaughterhouses. Specifically, the TPVCs and coliform counts were significantly higher in the samples from abattoir B compared to those from abattoir A (*p* < 0.01 in both cases). Conversely, the TMVCs were significantly higher in the samples from abattoir A than in those from abattoir B (*p* < 0.01). The median TMVCs in both abattoirs surpassed the lower threshold of 3.5 log_10_ CFU/cm^2^, but did not exceed the upper limit of 5.0 log_10_ CFU/cm^2^, as established by EU Commission Regulation No. 2073/2005 on microbiological criteria for foodstuffs. On the other hand, the coliform counts for carcasses from both slaughterhouses were below the lower limit of 1.5 log_10_ set by the same regulation. All raw microbiological data on the beef carcasses for each animal are provided in Supplementary Table S1.

## 4. Discussion

Considering the gap in the existing literature, the present study aimed to thoroughly characterize the beef quality across different breeds, from whole carcasses to individual muscle fibers. We also assessed slaughter hygiene by conducting microbiological analyses on the beef carcasses. The results showed that the breed significantly influenced most of the studied traits, including tenderness, colour, chemical composition, and fatty acid profile, all of which indicate the overall quality of the meat produced.

Consumer purchasing decisions regarding meat are significantly influenced by colour. Generally, consumers prefer bright red beef, as they associate this colour with freshness. In contrast, pale or dark beef is often considered to be of lower quality and less appealing [32,33,34]. Research shows that several intrinsic and extrinsic factors can affect meat colour. Intrinsic factors are related to the genetic variations of the animals, while extrinsic factors are linked to production systems and management practices [35]. Specifically, meat lightness could be affected by changes in the ultimate pH, heme iron, and intramuscular fat content, while meat redness is strongly associated with the metmyoglobin content [13,17,36]. Additionally, regarding extrinsic factors, meat colour is highly influenced by pre-mortem stress [35]. In our research, the breed significantly affected all of the measured colour parameters. Specifically, the beef produced from the AA and HO breeds was darker than the LI beef. Similarly to our study, Cuvelier et al. [37] reported that the Aberdeen Angus breed produced darker meat compared to the Belgian Blue and Limousin breeds. In contrast, the study by King et al. [38] showed that beef from AA crossbred steers had higher L* values than those of LI beef. However, other studies have reported no significant differences in the meat lightness (L*) between breeds [15,39]. Additionally, in our study, the CR males (Charolais x Limousin crosses) produced the reddest beef compared to the AA and HO breeds. Accordingly, another study revealed that Charolais and Limousin beef exhibited higher (*p* < 0.05) a* values than the Angus breed [38]. It is worth mentioning the results of the Cuvelier et al. [37] study, indicating that between d2 and d8 after slaughter, the meat redness decreased significantly in AA beef, while it slightly increased in LI beef. On the contrary, in one study [40], the meat redness did not differ significantly between the AA and LI breeds, while other authors reported no significant effects of the breed on meat redness [15,17]. Regarding the meat yellowness, AA beef exhibited the highest b* values, while CR beef had the lowest. Conversely, other studies suggest that meat yellowness is not affected by breed [15,17,39,40]. Differences in feeding practices, animal management, and study design protocols applied across countries could explain the variability in meat colour parameters observed between studies.

Among the meat quality characteristics, beef texture—particularly the hardness—is considered one of the most important factors influencing consumer purchasing decisions. Research has shown that consumers are willing to pay higher prices for premium cuts with greater tenderness [17,41]. In our study, beef produced by AA and CR males was more tender than LI beef. This finding may be attributed to the higher fat content observed in AA and CR beef compared to LI beef. Similarly, Bureš et al. [15] reported that the AA breed produced more tender beef compared to the HO breed. However, such findings were not corroborated by other studies, suggesting no significant differences in the beef tenderness between the LI and AA breeds [37,39,40]. Moreover, several studies have indicated that the breed has no significant effects on meat tenderness [17,42,43,44].

Furthermore, one significant factor affecting beef quality is the ultimate pH, mainly influenced by muscle glycogen concentrations. Specifically, the muscle glycogen levels decrease when animals are subjected to stressful conditions before and during slaughter [45]. This reduction leads to a lower production of lactic acid and higher pH values. Ultimate pH values greater than 5.8 have been associated with undesirable traits in beef, such as a darker colour, tougher texture, reduced shelf life, and poor flavour, all of which indicate a lower meat quality [46,47,48]. Similarly to other studies, our results showed that breed had no significant effects on the pH_24_, with all breeds exhibiting average pH_24_ values ranging from 5.5 to 5.6 [15,17,39,43].

Genetic variation among breeds is an important factor influencing the meat chemical composition [49]. The muscle lipid content varies between early- and late-maturing breeds due to differences in cattle tissue development and the distinct expression of fat regulatory genes [37,50]. Particularly, the HO breed tends to prioritize fat accumulation in internal stores rather than intramuscularly, as compared to the AA breed [51]. In contrast, the muscle protein content shows less variability among finishing cattle [52]. In our study, late-maturing LI males had the leanest meat, and early maturing AA males exhibited the highest fat content, with the HO breed having an intermediate value. Similar studies have shown significantly higher fat and protein levels in AA and LI breeds, respectively [37,39,49]. Besides the fat and protein content, total collagen is also taken into account when assessing beef’s chemical composition. Collagen is a group of connective tissue proteins supporting skeletal muscles, mainly influenced by breed, age, muscle, sex, and feeding [53,54]. In our study, the AA beef exhibited the highest collagen content compared to the HO and LI beef. Similarly, in one study, early maturing breeds were found to have a higher total collagen content compared to late maturing breeds [55].

The beef nutritional value is highly associated with the fatty acid (FA) profile, which is influenced by several factors such as breed, nutrition, age, and sex [56]. The FA profile is a key factor for consumers when purchasing beef; the increased consumption of SFAs has been linked with adverse health implications, while unsaturated FAs (MUFAs and PUFAs) provide cardiovascular benefits [50]. In our study, the AA beef contained significantly higher MUFA and lower SFA concentrations, whereas the HO beef exhibited the opposite trend; however, breed had no significant effects on the PUFA levels. Accordingly, previous studies have also reported higher MUFA concentrations in AA beef, which may be attributed to its higher intramuscular fat (IMF) content [50,57]. Oleic acid, the primary MUFA in beef, was abundant in breeds selected for high IMF [58]. Similarly, Garcia et al. [56] reported no significant differences in the beef PUFA concentration between AA and HO males.

The minimum Feret diameter is a reliable measurement of the individual muscle fiber diameter, as it is not affected by the sectioning angle and orientation errors [59]. Authors have suggested a strong correlation between a large muscle fiber diameter and improved meat yield and tenderness, highlighting its significance in the meat production sector [60]. To the best of our knowledge, this is the first study investigating breeds’ effects on the muscle fiber diameter, by applying this methodology; however, the *Longissimus dorsi* minimum Feret diameter did not differ significantly between the breeds. Given the existing gap in the literature, further research is needed to support our findings.

Regarding the slaughter hygiene assessment, our study revealed that the median TMVCs in both examined abattoirs exceeded the lower limit of 3.5 log_10_ CFU/cm^2^ but remained below the upper limit of 5.0 log_10_ CFU/cm^2^ set by EU Commission Regulation No. 2073/2005 on microbiological criteria for foodstuffs [28]. Notable differences in the microbial counts for the beef carcasses were observed between the two abattoirs. More specifically, abattoir A exhibited significantly higher TMVCs, whereas abattoir B showed significantly higher TPVCs and coliform counts. The elevated coliform counts in abattoir B indicate greater fecal contamination, likely occurring during critical processing stages such as dehiding and evisceration, where *Enterobacteriaceae* contamination is most common [11]. This observation can be justified by the higher slaughter throughput in abattoir B, which reflects a more intensive slaughter operation prone to slaughtering faulty practices, such as hide-to-carcass contact and gut rupture. The heavy workload and time pressure may also contribute to delayed or inefficient carcass chilling, favoring the growth of psychrophilic bacteria. Inadequate cleaning of chilling systems can also allow these cold-tolerant microorganisms to persist and form biofilms, leading to cross-contamination. Conversely, the higher TMVCs in abattoir A can result from inadequate hygiene practices attributable to the actions of slaughterhouse personnel during the warm stages of slaughter, such as dressing and handling [61]. The significant variations in microbial counts observed in the present study highlight inconsistencies in slaughter hygiene practices and the lack of uniform hygienic conditions throughout the process.

Several other European studies have reported TMVCs below the threshold of 3.5 log_10_ CFU/cm^2^, along with lower coliform counts compared to the present study, suggesting superior hygienic and slaughtering conditions in those abattoirs [62,63]. Conversely, studies from developing countries frequently report significantly higher TMVCs and coliform counts, often exceeding the upper limits set by European regulations [64,65]. Regardless of geographical location, all studies highlight substantial variability in microbial contamination levels among different abattoirs, reflecting differences in hygiene measures and slaughtering practices. Camargo et al. [61] found that microbial counts for beef carcasses varied widely across slaughterhouses in different Brazilian states, with some facilities maintaining TMVCs and coliform counts below the European regulatory threshold, while others exceeded it. Beyond the sampling techniques and the specific sites on the carcass selected for sampling, variations in microbial counts across studies can largely be attributed to differences in slaughtering procedures and hygienic practices. Critical steps such as dehiding, evisceration, and washing play a key role in determining the level of microbial contamination on beef carcasses across different abattoirs. To improve slaughter hygiene conditions, the stricter implementation of good manufacturing practices during these stages is essential, particularly to prevent hide-to-carcass contact and minimize the risk of gut rupture during evisceration. Moreover, adherence to basic hygiene principles, including effective hand hygiene, the use of clean protective equipment, and regular sanitation of tools and surfaces, is important to reduce the risk of cross-contamination. Regular training of slaughterhouse personnel can further enhance compliance with hygiene protocols. These targeted operational measures can help reduce microbial loads on beef carcasses and ensure better alignment with regulatory requirements.

This comprehensive study provides data on meat quality produced in Greece, with significant variations between breeds observed. The quality traits examined included the meat colour, texture, and chemical composition, which are critical in influencing consumer preferences. In our study, the CR beef had a richer red colour, often associated with freshness and quality. Furthermore, the AA and CR breeds produced more tender and juicy meat, appealing to consumers who prioritize these characteristics in their meat choices. Higher protein levels were also noted for LI and HO beef, addressing the diverse nutritional preferences and dietary needs of consumers. These differences in meat quality traits could significantly influence consumers’ purchasing decisions. Moreover, breeders could align with consumer preferences for traits such as tenderness, colour, and flavour, ultimately impacting market demand and pricing. In addition, the significant differences observed in the microbial counts between the two slaughterhouses highlight that slaughter hygiene plays a crucial role in meat quality. Overall, these findings underscore both the importance of breed selection in aligning meat production with market demands and the critical role of slaughter hygiene in ensuring beef quality, safety, and extended shelf life. Future research, including larger sample sizes of both foreign and local breeds, could provide further insights into the quality of meat produced in Greece.

## 5. Conclusions

The results of the present study demonstrate significant variations in most beef carcass quality traits among different breeds. The Charolais x Limousin crosses produced the reddest meat, which is more appealing to consumers in terms of colour. The AA and CR beef exhibited greater tenderness, potentially improving the overall eating quality for consumers. LI and HO beef could be more appealing to consumers prioritizing higher meat protein content, whereas AA beef is better suited for premium meat markets targeting taste and texture. Breeders have a valuable chance to align with consumer preferences by focusing on desirable traits that influence market demand. Adopting effective management practices in breed selection could lead to enhanced meat quality and higher product prices. Given the significant impact of slaughter hygiene on beef quality traits, improved slaughtering procedures and stricter adherence to good hygiene practices in slaughterhouses, particularly the consistent application of good manufacturing practices and personal hygiene measures, are necessary to reduce the microbial counts of beef carcasses and ensure compliance with European Regulations. Overall, this study significantly contributes to Greek meat science by providing extensive data on meat quality from whole carcasses to individual fibers. Given the scarcity of such research, the findings address an important knowledge gap and greatly benefit breeders, consumers, and the entire meat industry. Future studies that include larger samples of breeds and slaughterhouses could further boost these advantages, suggesting effective management practices for producing high-quality products and meeting consumer demands.

## Figures and Tables

**Table 1:** Descriptive statistics of carcass and beef quality traits of Aberdeen Angus (AA), Holstein (HO), Limousin (LI), and crossbred (CR) males.

Traits	Aberdeen Angus		Limousin		Holstein		Crossbred	
	N	Mean (±SD)	N	Mean (±SD)	N	Mean (±SD)	N	Mean (±SD)
pH	34	5.5 (0.26)	39	5.6 (0.10)	42	5.6 (0.14)	38	5.6 (0.20)
Lightness—L*	38	38.2 (2.09)	39	40.1 (2.87)	40	37.7 (2.29)	38	38.9 (2.09)
Redness—a*	38	19.5 (2.17)	39	21.3 (2.09)	40	17.6 (1.78)	38	22.2 (1.70)
Yellowness—b*	38	10.2 (1.53)	39	7.0 (1.84)	40	7.8 (2.23)	38	5.4 (1.91)
Chroma	38	21.9 (1.89)	39	22.5 (2.05)	40	19.3 (2.02)	38	22.9 (1.99)
Hue angle	38	0.5 (0.04)	39	0.3 (0.08)	40	0.4 (0.10)	38	0.2 (0.07)
Hardness 1 (g)	35	1019.7 (845.53)	24	1712.2 (835.81)	36	1130.4 (625.75)	35	826.4 (420.18)
Hardness 2 (g)	36	862.9 (178.80)	26	1448.2 (692.11)	37	867.1 (470.25)	35	647.3 (340.75)
Springiness	38	0.8 (0.08)	39	0.9 (0.13)	39	0.7 (0.08)	38	0.7 (0.08)
Cohesiveness	38	0.5 (0.10)	39	0.5 (0.11)	41	0.5 (0.09)	38	0.5 (0.05)
Chewiness (g)	38	451.0 (341.51)	30	775.8 (388.33)	37	454.1 (277.82)	37	348.2 (255.07)
Protein (%)	37	20.2 (1.22)	33	22.8 (0.97)	41	22.1 (0.98)	N/A	N/A
Fat (%)	36	6.6 (2.70)	33	1.7 (0.37)	40	2.7 (0.91)	N/A	N/A
Collagen (%)	35	2.2 (0.48)	33	1.3 (0.19)	40	1.4 (0.38)	N/A	N/A
Feret’s diameter (μm)	N/A	N/A	31	30.6 (2.54)	17	31.5 (2.75)	32	29.8 (4.93)
Myristic acid (C14:0)	37	3.1 (0.41)	3	3.5 (0.35)	23	3.1 (0.46)	N/A	N/A
Myristoleic acid (C14:1)	37	0.9 (0.25)	3	0.4 (0.24)	17	0.5 (0.21)	N/A	N/A
Pentadecanoic acid (C15:0)	36	0.4 (0.07)	3	0.5 (0.03)	15	0.4 (0.97)	N/A	N/A
Palmitic acid (C16:0)	37	25.4 (2.03)	3	29.7 (1.54)	23	27.6 (1.80)	N/A	N/A
Palmitoleic acid (C16:1)	37	5.0 (0.79)	3	3.7 (0.45)	23	3.7 (0.79)	N/A	N/A
Heptadecanoic acid (C17:0)	37	1.1 (0.21)	3	0.9 (0.09)	23	0.9 (0.16)	N/A	N/A
Cis-10 Heptadecenoic acid (C17:1)	32	1.0 (0.25)	3	0.4 (0.07)	17	0.5 (0.18)	N/A	N/A
Stearic acid (C18:0)	37	12.2 (2.31)	3	19.3 (1.77)	23	20.1 (3.31)	N/A	N/A
Elaidic acid (C18:1n9t)	37	1.3 (1.1)	3	1.8 (0.34)	19	0.9 (0.60)	N/A	N/A
Oleic acid (C18:1n9c)	37	47.0 (2.92)	3	36.7 (2.73)	23	39.6 (3.15)	N/A	N/A
Linoleic acid (C18:2n6c)	37	1.7 (0.58)	3	0.2 (0.03)	23	2.2 (0.67)	N/A	N/A
Saturated fatty acids	37	42.4 (3.98)	3	54.1 (3.34)	23	52.3 (3.99)	N/A	N/A
Monounsaturated fatty acids	37	55.2 (3.58)	3	43.3 (2.28)	23	45.2 (3.80)	N/A	N/A
Polyunsaturated fatty acids	37	2.3 (0.67)	3	2.6 (0.13)	23	2.5 (0.70)	N/A	N/A
Total mesophilic viable counts (TMVCs, log_10_ CFU/cm^2^)	N/A	N/A	34	3.5 (0.72)	22	3.9 (0.70)	21	3.9 (0.94)
Total psychrophilic viable counts (TPVCs, log_10_ CFU/cm^2^)	N/A	N/A	34	3.7 (1.0)	22	3.1 (0.76)	21	1.9 (1.74)
Coliform counts (log_10_ CFU/cm^2^)	N/A	N/A	34	1.5 (0.76)	22	0.8 (0.93)	21	0.7 (0.88)

N: number of observations, SD: standard deviation, N/A: not applicable.

**Table 2:** One-way ANOVA results for colour parameters (lightness—L*, redness—a*, yellowness—b*, chroma) of Aberdeen Angus (AA), Limousin (LI), Holstein (HO), and crossbred (CR) males.

	Breed								
Trait	AA	LI	HO	CR					
	Mean (±SD)	Mean (±SD)	Mean (±SD)	Mean (±SD)	Sum of Squares	df	Mean Square	F	*p*-Value
Lightness—L*	38.2 ^b^ (2.09)	40.1 ^a^ (2.87)	37.7 ^c^ (2.29)	38.9 ^abc^ (2.09)	126.60	3	42.20	5.86	**<0.001**
Redness—a*	19.5 ^b^ (2.17)	21.3 ^a^ (2.09)	17.6 ^c^ (1.78)	22.2 ^a^ (1.70)	492.30	3	164.12	43.45	**<0.001**
Yellowness—b*	10.2 ^a^ (1.53)	7.0 ^b^ (1.84)	7.8 ^b^ (2.23)	5.4 ^c^ (1.91)	467.50	3	155.82	43.02	**<0.001**
Chroma	21.9 ^a^ (1.89)	22.5 ^a^ (2.05)	19.3 ^b^ (2.02)	22.9 ^a^ (1.99)	309.30	3	103.09	26.06	**<0.001**

^a,b,c^ Mean values not sharing any common letter are significantly different according to Tukey’s tests (*p* < 0.05).

**Table 3:** Kruskal–Wallis test results for texture parameters (Hardness 1 (g), Hardness 2 (g), Springiness, Cohesiveness, Chewiness (g)), meat pH, and hue angle for Aberdeen Angus (AA), Limousin (LI), Holstein (HO), and crossbred (CR) males.

	Breed								
Trait	AA		LI		HO		CR		
	Median (IQR)	Mean Rank	Median (IQR)	Mean Rank	Median (IQR)	Mean Rank	Median (IQR)	Mean Rank	*p*-Value
Hardness 1 (g)	683.8 (1061.76)	63.7 ^bc^	1611.0 (1217.0)	111.0 ^a^	1011.3 (937.52)	80.5 ^abc^	730.9 (717.79)	59.6 ^bc^	**<0.001**
Hardness 2 (g)	552.9 (911.52)	63.6 ^b^	1424.1 (1081.20)	113.0 ^a^	765.3 (692.47)	78.0 ^b^	593.5 (581.67)	61.1 ^b^	**<0.001**
Springiness	0.8 (0.13)	85.1 ^ab^	0.9 (0.19)	106.0 ^a^	0.7 (0.10)	71.7 ^bc^	0.7 (0.11)	53.6 ^bc^	**<0.001**
Cohesiveness	0.5 (0.12)	93.9 ^a^	0.5 (0.13)	60.4 ^b^	0.5 (0.10)	82.3 ^ab^	0.5 (0.05)	79.6 ^ab^	**0.013**
Chewiness (g)	314.2 (583.43)	64.4 ^b^	927.2 (782.08)	111.0 ^a^	382.9 (328.73)	80.8 ^b^	297.6 (251.92)	58.6 ^b^	**<0.001**
pH	5.6 (0.14)	80.0 ^a^	5.6 (0.07)	74.1 ^a^	5.6 (0.18)	85.9 ^a^	5.6 (0.10)	75.3 ^a^	0.472
Hue angle	0.5 (0.04)	124.0 ^a^	0.3 (0.07)	60.70 ^c^	0.4 (0.16)	98.10 ^b^	0.2 (0.12)	31.2 ^d^	**<0.001**

^a,b,c,d^ Mean ranks not sharing any common letter are significantly different according to Dunn’s tests (*p* < 0.05).

**Table 4:** Kruskal–Wallis test results for meat chemical composition by Limousin (LI), Aberdeen Angus (AA), and Holstein (HO) breeds.

	Breed						
Trait	AA		LI		HO		
	Median (IQR)	Mean Rank	Median (IQR)	Mean Rank	Median (IQR)	Mean Rank	*p*-Value
Protein (%)	20.3 (1.70)	25.80 ^b^	22.9 (1.45)	80.74 ^a^	22.3 (1.50)	63.34 ^a^	**<0.001**
Fat (%)	6.1 (3.50)	89.33 ^a^	1.6 (0.55)	22.47 ^c^	2.6 (1.10)	50.94 ^b^	**<0.001**
Collagen (%)	2.1 (0.60)	88.34 ^a^	1.4 (0.40)	35.62 ^b^	1.4 (0.45)	40.46 ^b^	**<0.001**

^a,b,c^ Mean ranks not sharing any common letter are significantly different according to Dunn’s tests (*p* < 0.05).

**Table 5:** Mann–Whitney U test results for meat fatty acid profile by Aberdeen Angus (AA) and Holstein (HO) breeds.

	Breed						
Trait	AA			HO			
	n	Median	IQR	n	Median	IQR	*p*-Value
Myristic acid (C14:0)	37	3.1	0.64	23	3.2	0.66	0.911
Myristoleic acid (C14:1)	37	0.9	0.34	17	0.5	0.32	**<0.001**
Pentadecanoic acid (C15:0)	37	0.4	0.12	15	0.3	0.10	0.108
Palmitic acid (C16:0)	37	25.0	2.77	23	27.6	1.85	**<0.001**
Palmitoleic acid (C16:1)	37	4.8	1.15	23	3.6	0.69	**<0.001**
Heptadecanoic acid (C17:0)	37	1.1	0.34	23	0.9	0.17	**0.003**
Cis-10 Heptadecenoic acid (C17:1)	32	1.0	0.33	17	0.4	0.25	**<0.001**
Stearic acid (C18:0)	37	11.9	3.16	23	20.2	4.32	**<0.001**
Elaidic acid (C18:1n9t)	37	1.0	0.67	19	0.8	0.42	0.073
Oleic acid (C18:1n9c)	37	47.6	4.04	23	40.0	5.07	**<0.001**
Linoleic acid (C18:2n6c)	37	1.7	0.55	23	2.2	0.80	**<0.001**
Saturated fatty acids	37	41.4	4.78	23	51.6	5.41	**<0.001**
Monounsaturated fatty acids	37	55.9	4.22	23	45.9	5.25	**<0.001**
Polyunsaturated fatty acids	37	2.3	0.76	23	2.6	0.91	0.088

**Table 6:** Results of the Mann–Whitney U test for microbial counts for carcasses from the two examined abattoirs.

	Abattoir A **			Abattoir B ***			
	n	Median	IQR	n	Median	IQR	*p*-Value
TMVCs * (log_10_ CFU/cm^2^)	22	4.3	0.10	55	3.6	1.04	**0.004**
TPVCs * (log_10_ CFU/cm^2^)	22	1.7	4.20	55	3.3	1.18	**0.006**
Coliform counts (log_10_ CFU/cm^2^)	22	0.0	1.08	55	1.3	1.57	**0.004**

* TMVCs = total mesophilic viable counts, TPVCs = total psychrophilic viable counts. ** In abattoir A, the 22 collected samples consisted of 21 crossbred carcasses and 1 Limousin beef carcass; *** in abattoir B, the 55 collected samples consisted of 33 Limousin and 22 Holstein beef carcasses.

## Data Availability

The original contributions presented in this study are included in the article/Supplementary Materials. Further inquiries can be directed to the corresponding author.

## Supplementary Materials

The following supporting information can be downloaded at https://www.mdpi.com/article/10.3390/foods14101776/s1: Dataset S1: Total dataset of beef carcass quality traits.
